# Supplementary material for: The Evolution and Characterization of the RNA Interference Pathways in Lophotrochozoa
Source: Genome Biol Evol. 2024 May 7;16(5):evae098. doi: 10.1093/gbe/evae098 (PMC11114477; doi:10.1093/gbe/evae098)
Supplement: evae098_Supplementary_Data [file evae098_supplementary_data.zip › Supplementary_materials.zip!/selected_miRNAs/NoGe_selected_miRNAs/mirdeep_pdf_files/NMRB01001254.1_Notospermus_geniculatus_strain_Ushimado_scaffold1254,_whole_genome_shotgun_sequence_26716.pdf]

Provisional ID	:	NMRB01001254.1_Notospermus_geniculatus_strain_Ushimado_scaffold1254,_whole_genome_shotgun_sequence_26716
Score total	:	88862.4
Score for star read(s)	:	3.9
Score for read counts	:	88854
Score for mfe	:	1.5
Score for randfold	:	0
Score for cons. seed	:	3
Total read count	:	174295
Mature read count	:	174095
Loop read count	:	0
Star read count	:	200

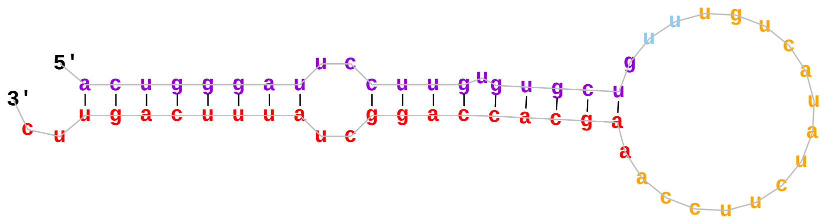

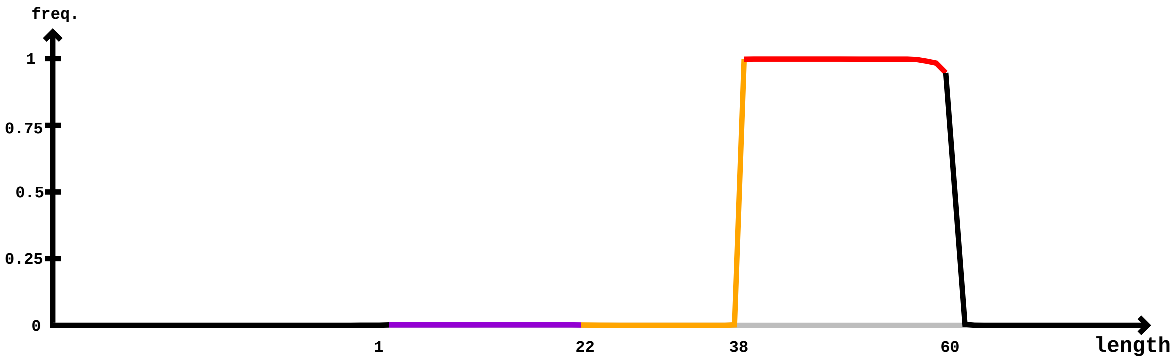

Star Mature

```
5'- agccagaacagagcugcugaaccuauccaguggacugggauuccuugugugcuguuugucauaucuuccaaagcaccaggcuauuucaguucucugguugggagaauucgac -3'  obs
    agccagaacagagcugcugaaccuauccaguggacugggauuccuugugugcuguuugucauaucuuccaaagcaccaggcuauuucaguucucugguugggagaauucgac       exp
    (((.((.......)))))...((((.((((.((((((((((..((((.(((((.................)))))))))..)))))))))).)))).))))...........   reads  mm  sample
    ..............................uggacugggauuccuugCgugcu...........................................................   1      1   Mm1
    ..............................uggacugggauuccuugugugcu...........................................................   12     0   Mm1
    ..............................uggacugggauuccuugGgugcu...........................................................   1      1   Mm1
    ..............................uggacugggaAuccuugugugcu...........................................................   1      1   Mm1
    ..............................uggacugggauuccuugugugcuU..........................................................   6      1   Mm1
    .................................acugggauuccuuguguAcuguuu.......................................................   1      1   Mm1
    .................................acugggauuccuuguguCcuguuu.......................................................   1      1   Mm1
    .................................acugggauuccuugugugcuguuu.......................................................   15     0   Mm1
    .................................acugggauuccuugugugcuguuug......................................................   10     0   Mm1
    .................................acugggauuccuugugugcuguuugucauaucuucc...........................................   1      0   Mm1
    ..........................................................ucauaucuuccaaagcaUcaggcu..............................   1      1   Mm1
    ..........................................................ucauaucuuccaaagcaccaggcu..............................   4      0   Mm1
    ..................................................................uccaaagcaccaggcuauuucaguucuc..................   5      0   Mm1
    .....................................................................aaagcaccaggcuauuucaguuc....................   10     0   Mm1
    ......................................................................aagcaccaggcuauuucU........................   6      1   Mm1
    ......................................................................aagcaccaggcuCuuuca........................   1      1   Mm1
    ......................................................................aagcaccaggcuauuuca........................   69     0   Mm1
    ......................................................................aagcCccaggcuauuucag.......................   1      1   Mm1
    ......................................................................aagcaccaggcuauuucag.......................   32     0   Mm1
    ......................................................................aagcaccaggcuauuucaU.......................   1      1   Mm1
    ......................................................................aagcaccaggcuauuuAagu......................   2      1   Mm1
    ......................................................................aagcaccaggcuauuucagu......................   154    0   Mm1
    ......................................................................aagcaccaggcuauuucUgu......................   1      1   Mm1
    ......................................................................aagcaccaggcuauuuGagu......................   2      1   Mm1
    ......................................................................aagcaccaggcuauuuGaguu.....................   1      1   Mm1
    ......................................................................aagcaccaggcuaCuucaguu.....................   1      1   Mm1
    ......................................................................aagcaccaggcuauuucaguA.....................   7      1   Mm1
    ......................................................................aagcaccaggcuauuucaguu.....................   722    0   Mm1
    ......................................................................Uagcaccaggcuauuucaguu.....................   2      1   Mm1
    ......................................................................aagcaccaggGuauuucaguu.....................   1      1   Mm1
    ......................................................................aagcaccaggcuauuuAaguu.....................   3      1   Mm1
    ......................................................................aagcaccaggcuauuucCguu.....................   7      1   Mm1
    ......................................................................aagAaccaggcuauuucaguu.....................   1      1   Mm1
```

```
                               Star                           Mature
agccagaacagagcugcugaaccuauccaguggacugggauuccuugugugcuguuugucauaucuuccaaagcaccaggcuauuucaguucucugguugggagaauucgac
......................................................................Nagcaccaggcuauuucaguu.....................    1      1    Mm1
......................................................................aagcaccaggcuauuucUguu.....................    2      1    Mm1
......................................................................aagcaccaggcuaAuucaguu.....................    1      1    Mm1
......................................................................aagcaccaggAuauuucaguu.....................    1      1    Mm1
......................................................................aagcaccaggUuauuucaguu.....................    2      1    Mm1
......................................................................aagcaccaggcuauuucaguG.....................    1      1    Mm1
......................................................................aagcaccaggcuauuuUaguu.....................    3      1    Mm1
......................................................................aagcaccaggcuauuucaguAc....................    2      1    Mm1
......................................................................aagcaccaggcuauuucCguuc....................    20     1    Mm1
......................................................................aagcacAaggcuauuucaguuc....................    1      1    Mm1
......................................................................aagcacUaggcuauuucaguuc....................    2      1    Mm1
......................................................................aagcaccaggcuauuucGguuc....................    19     1    Mm1
......................................................................aagcaccaggcuauuucUguuc....................    27     1    Mm1
......................................................................aagcCccaggcuauuucaguuc....................    1      1    Mm1
......................................................................aagcaccaggcuaGuucaguuc....................    1      1    Mm1
......................................................................Uagcaccaggcuauuucaguuc....................    7      1    Mm1
......................................................................aagcaccaggcuauGucaguuc....................    1      1    Mm1
......................................................................aagAaccaggcuauuucaguuc....................    2      1    Mm1
......................................................................aagcaccaggcuauAucaguuc....................    1      1    Mm1
......................................................................aagcaccaggcuauuucaguuU....................    19     1    Mm1
......................................................................aaAcaccaggcuauuucaguuc....................    1      1    Mm1
......................................................................aagcaccGggcuauuucaguuc....................    2      1    Mm1
......................................................................aUgcaccaggcuauuucaguuc....................    2      1    Mm1
......................................................................aagcaccaggUuauuucaguuc....................    5      1    Mm1
......................................................................aagcaccaggcuaAuucaguuc....................    3      1    Mm1
......................................................................aagcaccagCcuauuucaguuc....................    1      1    Mm1
......................................................................aagcaccUggcuauuucaguuc....................    1      1    Mm1
......................................................................Nagcaccaggcuauuucaguuc....................    9      1    Mm1
......................................................................aagcaccaggcuauuucaUuuc....................    2      1    Mm1
......................................................................aagcaccagUcuauuucaguuc....................    2      1    Mm1
......................................................................aagcaccaggcuauuuUaguuc....................    20     1    Mm1
......................................................................Gagcaccaggcuauuucaguuc....................    1      1    Mm1
......................................................................aagcGccaggcuauuucaguuc....................    2      1    Mm1
......................................................................aagcaccaggcuauuuGaguuc....................    15     1    Mm1
......................................................................aagcaccaggcGauuucaguuc....................    1      1    Mm1
......................................................................aagcaccaggcuauCucaguuc....................    2      1    Mm1
......................................................................aagGaccaggcuauuucaguuc....................    2      1    Mm1
......................................................................aagcaccaggGuauuucaguuc....................    6      1    Mm1
......................................................................aagcaccaggcuauuucaguuc....................    7960   0    Mm1
......................................................................aaCcaccaggcuauuucaguuc....................    1      1    Mm1
......................................................................aagcaNcaggcuauuucaguuc....................    2      1    Mm1
......................................................................aagcaccaggcuauuucaguuG....................    1      1    Mm1
......................................................................aaUcaccaggcuauuucaguuc....................    3      1    Mm1
......................................................................aagcaccaggAuauuucaguuc....................    9      1    Mm1
......................................................................aGgcaccaggcuauuucaguuc....................    2      1    Mm1
......................................................................aagcaccaggcCauuucaguuc....................    1      1    Mm1
......................................................................aagcaccaggcuaCuucaguuc....................    1      1    Mm1
......................................................................aagcaccaggcuauuuAaguuc....................    29     1    Mm1
......................................................................aagcaccaggcuauuCcaguuc....................    1      1    Mm1
......................................................................Cagcaccaggcuauuucaguucu...................    1      1    Mm1
......................................................................aagcaccaggcuauuuUaguucu...................    1      1    Mm1
......................................................................aagcaccaggUuauuucaguucu...................    1      1    Mm1
......................................................................aagcaccaggcuauuucaguucA...................    455    1    Mm1
......................................................................aagcaccaggcuauuucaguucG...................    10     1    Mm1
......................................................................Nagcaccaggcuauuucaguucu...................    1      1    Mm1
......................................................................aagcaccaggcuauuucUguucu...................    1      1    Mm1
......................................................................aagcaccaggcuauuucaguucu...................    394    0    Mm1
......................................................................aagcaccaggcuauuuGaguucu...................    2      1    Mm1
......................................................................aagcaccaggAuauuucaguucu...................    1      1    Mm1
......................................................................aagcaccaggcuauuuAaguucu...................    1      1    Mm1
......................................................................aagcaccaggcuauuucaguucC...................    34     1    Mm1
......................................................................aGgcaccaggcuauuucaguucu...................    1      1    Mm1
......................................................................aagcaccaggcuauuucGguucu...................    1      1    Mm1
......................................................................aagcaccaggcuauuucaguucAc..................    2      1    Mm1
......................................................................aagcaccaggcuauuucaguucuU..................    9      1    Mm1
......................................................................aagcaccaggcuauuucaguucuUu.................    1      1    Mm1
.......................................................................agcaccaggcuauuucaguu.....................    7      0    Mm1
.......................................................................agcaccaggcuauuucaguuc....................    11     0    Mm1

........cagagcugcugaaccuauccagugg...............................................................................    2      0    Mm2
```

```
                                 Star                                Mature
agccagaacagagcugcugaaccuauccaguggacugggauuccuugugugcuguuugucauaucuuccaaagcaccaggcuauuucaguucucugguugggagaauucgac
..............................uggacugggauuccuugugugc............................................................ 1 0 Mm2
..............................uggacugggauuccuugugugcG........................................................... 1 1 Mm2
..............................uggacugggauuccuugugugcu........................................................... 4 0 Mm2
.................................acugggauuccuugugugcug.......................................................... 3 0 Mm2
.................................acugggauuccuugugugcuguu........................................................ 5 0 Mm2
.................................acugggauuccuuguguAcuguuu....................................................... 1 1 Mm2
.................................acugggauuccuugugugcuguuu....................................................... 1 0 Mm2
.................................acugggauuccuugugugcuguuug...................................................... 3 0 Mm2
.................................acugggauuccuugugugcuguuugA..................................................... 2 1 Mm2
.................................acugggauuccuugugugcuguuugucau.................................................. 1 0 Mm2
.................................acugggauuccuugugugcuguuugucauaucuucca.......................................... 1 0 Mm2
...........................................cuugugugcuguuugucauaucuucca.......................................... 1 0 Mm2
..........................................................ucauaucuuccaaagcaccaggc............................... 4 0 Mm2
..........................................................ucauaucuuccaaagcaccaggcu.............................. 6 0 Mm2
..........................................................ucauaucuuccaaagcaccaggcuauuucaguucu................... 1 0 Mm2
..........................................................ucauaucuuccaaagcaccaggcuauuucGguucu................... 1 1 Mm2
.....................................................................aaagcaccaggcuauuuc......................... 1 0 Mm2
.....................................................................aaagcaccaggcuauuucagu...................... 1 0 Mm2
.....................................................................aaagcaccaggcuauuucaguu..................... 2 0 Mm2
.....................................................................aaagcaccaggcuauuucaguuc.................... 50 0 Mm2
.....................................................................aaagcaccagAcuauuucaguuc.................... 1 1 Mm2
.....................................................................Caagcaccaggcuauuucaguuc.................... 12 1 Mm2
.....................................................................aaagcaccaggcuauuucaguuU.................... 2 1 Mm2
.....................................................................aaagcaccaggcuauuucaguucu................... 10 0 Mm2
......................................................................aagcaccaggcuauuucU........................ 2 1 Mm2
......................................................................aagcaccaggcuauuuca........................ 47 0 Mm2
......................................................................aagcaccaggcuauuuUa........................ 2 1 Mm2
......................................................................aagcaccaggcuauuucG........................ 1 1 Mm2
......................................................................aagcaccaggcuauuucaA....................... 4 1 Mm2
......................................................................aagcaccaggcuauuucag....................... 104 0 Mm2
......................................................................aagcaccaggcuauuucaU....................... 1 1 Mm2
......................................................................aagcacUaggcuauuucag....................... 1 1 Mm2
......................................................................aagcaccaggcuauuuAag....................... 1 1 Mm2
......................................................................aagcaAcaggcuauuucagu...................... 1 1 Mm2
......................................................................aagcaccaggcuauuucCgu...................... 4 1 Mm2
......................................................................aagcaccaggcuauuucagA...................... 1 1 Mm2
......................................................................aagcaccaggcuauuucagu...................... 196 0 Mm2
......................................................................aagcaccaggcuauuucaguG..................... 1 1 Mm2
......................................................................aagcaccaggcuauuucCguu..................... 5 1 Mm2
......................................................................aagcaccaggcuauuucaguC..................... 1 1 Mm2
......................................................................aagcaccaggcuauuuUaguu..................... 3 1 Mm2
......................................................................aagcacUaggcuauuucaguu..................... 1 1 Mm2
......................................................................aagcaccaggcuauuucagCu..................... 1 1 Mm2
......................................................................aGgcaccaggcuauuucaguu..................... 1 1 Mm2
......................................................................aagcaccaggGuauuucaguu..................... 1 1 Mm2
......................................................................aaCcaccaggcuauuucaguu..................... 1 1 Mm2
......................................................................aagcaccaggcuauuucGguu..................... 3 1 Mm2
......................................................................aagUaccaggcuauuucaguu..................... 1 1 Mm2
......................................................................aagcaccaggcuauuuAaguu..................... 6 1 Mm2
......................................................................aagcaccaggAuauuucaguu..................... 2 1 Mm2
......................................................................aagcaccaggcuauuuGaguu..................... 3 1 Mm2
......................................................................aagcaUcaggcuauuucaguu..................... 2 1 Mm2
......................................................................aagcaccaggcuauuucaguu..................... 1398 0 Mm2
......................................................................aagcaccaggcuauuucaguA..................... 10 1 Mm2
......................................................................aagcaccaggcuauCucaguu..................... 1 1 Mm2
......................................................................aagcUccaggcuauuucaguu..................... 1 1 Mm2
......................................................................aagcaccaggcuauuucUguu..................... 8 1 Mm2
......................................................................aagcaccaggcuauuCcaguu..................... 1 1 Mm2
......................................................................aagUaccaggcuauuucaguuc.................... 4 1 Mm2
......................................................................aagcaccaggcuauuucagCuc.................... 6 1 Mm2
......................................................................aagcUccaggcuauuucaguuc.................... 2 1 Mm2
......................................................................aagcaccaggcuauuucaguuU.................... 203 1 Mm2
......................................................................aagcaccaggcuaCuucaguuc.................... 7 1 Mm2
......................................................................aagcaccGggcuauuucaguuc.................... 8 1 Mm2
......................................................................aagcaccaggcuauuucagAuc.................... 3 1 Mm2
......................................................................aagcacGaggcuauuucaguuc.................... 4 1 Mm2
......................................................................aagcaccaCgcuauuucaguuc.................... 1 1 Mm2
......................................................................aagcaccaggcuauuucUguuc.................... 138 1 Mm2
......................................................................aagcCccaggcuauuucaguuc.................... 2 1 Mm2
......................................................................aagcaccaggcuauuucaguuG.................... 1 1 Mm2
```

Star Mature

```
agccagaacagagcugcugaaccuauccaguggacugggauuccuugugugcuguuugucauaucuuccaaagcaccaggcuauuucaguucucugguugggagaauucgac
......................................................................Nagcaccaggcuauuucaguuc....................  22     1  Mm2
......................................................................aagcaccaggcuauCucaguuc....................  3      1  Mm2
......................................................................aagcacNaggcuauuucaguuc....................  8      1  Mm2
......................................................................Gagcaccaggcuauuucaguuc....................  9      1  Mm2
......................................................................Uagcaccaggcuauuucaguuc....................  15     1  Mm2
......................................................................aagcaccaggcuauuucaguuc....................  43690  0  Mm2
......................................................................aagcaccaggcuauuAcaguuc....................  7      1  Mm2
......................................................................aagcaccagAcuauuucaguuc....................  4      1  Mm2
......................................................................aagcaccCggcuauuucaguuc....................  4      1  Mm2
......................................................................aUgcaccaggcuauuucaguuc....................  7      1  Mm2
......................................................................aagcaccaggcuauGucaguuc....................  1      1  Mm2
......................................................................aagcaccaUgcuauuucaguuc....................  8      1  Mm2
......................................................................aagcaNcaggcuauuucaguuc....................  11     1  Mm2
......................................................................aagcaGcaggcuauuucaguuc....................  2      1  Mm2
......................................................................aaCcaccaggcuauuucaguuc....................  3      1  Mm2
......................................................................aagcGccaggcuauuucaguuc....................  3      1  Mm2
......................................................................aagcaccaggcuauuucaguCc....................  8      1  Mm2
......................................................................aagcaccUggcuauuucaguuc....................  14     1  Mm2
......................................................................aagcaccaggcuauuuUaguuc....................  114    1  Mm2
......................................................................aaAcaccaggcuauuucaguuc....................  16     1  Mm2
......................................................................aagcaccaggcuauuucaUuuc....................  6      1  Mm2
......................................................................aagcaccaggcuauuucaguAc....................  4      1  Mm2
......................................................................aagcaccaggcuaGuucaguuc....................  4      1  Mm2
......................................................................aagcaccagUcuauuucaguuc....................  3      1  Mm2
......................................................................aagAaccaggcuauuucaguuc....................  5      1  Mm2
......................................................................aagcaccagCcuauuucaguuc....................  2      1  Mm2
......................................................................aagcaccaggAuauuucaguuc....................  94     1  Mm2
......................................................................aagcaccaggcCauuucaguuc....................  4      1  Mm2
......................................................................aagcaccaggcuauuucCguuc....................  133    1  Mm2
......................................................................aGgcaccaggcuauuucaguuc....................  7      1  Mm2
......................................................................aagcaccaggcuauuucagGuc....................  2      1  Mm2
......................................................................aagcaccaggUuauuucaguuc....................  30     1  Mm2
......................................................................aagcacUaggcuauuucaguuc....................  12     1  Mm2
......................................................................aagcaccaggcAauuucaguuc....................  2      1  Mm2
......................................................................aagcaccaggcGauuucaguuc....................  3      1  Mm2
......................................................................aagcaccaggcuauuuAaguuc....................  191    1  Mm2
......................................................................aaUcaccaggcuauuucaguuc....................  17     1  Mm2
......................................................................aagcaccaggGuauuucaguuc....................  38     1  Mm2
......................................................................aagcaccaggcuUuuucaguuc....................  8      1  Mm2
......................................................................aagcaccaggcuauAucaguuc....................  2      1  Mm2
......................................................................aagcaccaggcuCuuucaguuc....................  2      1  Mm2
......................................................................aagcacAaggcuauuucaguuc....................  4      1  Mm2
......................................................................aagcaccaggcuauuucaguuA....................  14     1  Mm2
......................................................................aagcaccaggcuauuCcaguuc....................  9      1  Mm2
......................................................................aagcaccaAgcuauuucaguuc....................  4      1  Mm2
......................................................................aagcaccaggcuGuuucaguuc....................  5      1  Mm2
......................................................................aagcaUcaggcuauuucaguuc....................  12     1  Mm2
......................................................................aagcaccaggcuauuucGguuc....................  129    1  Mm2
......................................................................aCgcaccaggcuauuucaguuc....................  2      1  Mm2
......................................................................aagcaAcaggcuauuucaguuc....................  2      1  Mm2
......................................................................aagcaccaggcuauuuGaguuc....................  60     1  Mm2
......................................................................aagcaccaggcuauuucaAuuc....................  4      1  Mm2
......................................................................aagcaccaggcuauuucaguGc....................  2      1  Mm2
......................................................................Cagcaccaggcuauuucaguuc....................  3      1  Mm2
......................................................................aagcaccaggcuaAuucaguuc....................  14     1  Mm2
......................................................................aagcaccaggcuauuucaguucC...................  93     1  Mm2
......................................................................aagcaccGggcuauuucaguucu...................  1      1  Mm2
......................................................................aagcaccaggcuauuuUaguucu...................  11     1  Mm2
......................................................................aagcaccaUgcuauuucaguucu...................  1      1  Mm2
......................................................................aGgcaccaggcuauuucaguucu...................  1      1  Mm2
......................................................................aagcaccaggUuauuucaguucu...................  5      1  Mm2
......................................................................aagcaccaggcuauuucaguucA...................  1549   1  Mm2
......................................................................aagcaccaggcuauuucGguucu...................  7      1  Mm2
......................................................................aagcaccaggcuauuucCguucu...................  7      1  Mm2
......................................................................aaCcaccaggcuauuucaguucu...................  1      1  Mm2
......................................................................aagcaccaggcuauuucUguucu...................  8      1  Mm2
......................................................................aagcaccaggcuaCuucaguucu...................  1      1  Mm2
......................................................................aagcaccaggcuauuuAaguucu...................  5      1  Mm2
......................................................................aagcaccaggAuauuucaguucu...................  3      1  Mm2
......................................................................aagcGccaggcuauuucaguucu...................  1      1  Mm2
```

```
                                Star                                   Mature
agccagaacagagcugcugaaccuauccaguggacugggauuccuugugugcuguuugucauaucuuccaaagcaccaggcuauuucaguucucugguugggagaauucgac
......................................................................aagcaccaggcuauuCcaguucu...................   1     1   Mm2
......................................................................aagcaccaggcuauuucaguuUu...................   10    1   Mm2
......................................................................aagcaccaggcuauuucaguuAu...................   1     1   Mm2
......................................................................aagcaccaggcuauuucaguucG...................   15    1   Mm2
......................................................................aagcaccaggcuauCucaguucu...................   1     1   Mm2
......................................................................aagcaccaggcuauuucaguucu...................   2107  0   Mm2
......................................................................aagcaccagUcuauuucaguucu...................   1     1   Mm2
......................................................................aagcaccaggGuauuucaguucu...................   2     1   Mm2
......................................................................aagcaNcaggcuauuucaguucu...................   1     1   Mm2
......................................................................aagcaccaggcuauuucaguucuA..................   3     1   Mm2
......................................................................aagcaccaggcuauuucaguucuU..................   76    1   Mm2
......................................................................aagcaccaggcuauuucaguucAc..................   1     1   Mm2
......................................................................aagcaccaggcuauuucaguucuUu.................   16    1   Mm2
.......................................................................agcaccaggcuauuucagu......................   1     0   Mm2
.......................................................................agcaccaggcuauuucaguuc....................   16    0   Mm2
.......................................................................agcaccaggcuauuucaguucu...................   2     0   Mm2

..ccagaacagagcugcugaaccuauccagu.................................................................................   2     0   Fm1
..............................uggacugggauuccuugugugcu...........................................................   1     0   Fm1
.................................acugggauuccuugugugcug..........................................................   3     0   Fm1
.................................acugggauuccuugugugcGg..........................................................   1     1   Fm1
.................................acugggauuccuugugugcuguuu.......................................................   3     0   Fm1
...........................................cuugugugcuguuugucauaucuucca..........................................   4     0   Fm1
..........................................................ucauaucuuccaaagcaccaggcu..............................   1     0   Fm1
.....................................................................aaagcaccaggcuauuuAagu......................   1     1   Fm1
.....................................................................aaagcaccaggcuauuucagu......................   1     0   Fm1
.....................................................................aaagcaccaggcuauuucaguG.....................   1     1   Fm1
.....................................................................aaagcaccaggcuauuucaguu.....................   19    0   Fm1
.....................................................................Caagcaccaggcuauuucaguu.....................   4     1   Fm1
.....................................................................aaagcacGaggcuauuucaguu.....................   1     1   Fm1
.....................................................................Caagcaccaggcuauuucaguuc....................   18    1   Fm1
.....................................................................aaagcaccaggcuauuucaguuc....................   67    0   Fm1
.....................................................................aaagcaccaggcuauuuAaguuc....................   1     1   Fm1
.....................................................................Caagcaccaggcuauuucaguucu...................   5     1   Fm1
......................................................................aagcaccaggcuauuuAa........................   2     1   Fm1
......................................................................aagcaccaggcuauuuca........................   104   0   Fm1
......................................................................aagcaccaggcuauuuUag.......................   1     1   Fm1
......................................................................aGgcaccaggcuauuucag.......................   1     1   Fm1
......................................................................aagcaccaggcuauuucUg.......................   1     1   Fm1
......................................................................aagcaccaggcuauuucaU.......................   2     1   Fm1
......................................................................aagcaccaggcuauuucaA.......................   1     1   Fm1
......................................................................aagcaccaggcuauuucag.......................   137   0   Fm1
......................................................................Nagcaccaggcuauuucagu......................   1     1   Fm1
......................................................................aagcaccaggcuauuucagu......................   503   0   Fm1
......................................................................aagcaccaggGuauuucagu......................   1     1   Fm1
......................................................................aagcaccaggcuauuucagA......................   6     1   Fm1
......................................................................aagcaccaggcuauuucCgu......................   1     1   Fm1
......................................................................aagcaccaggcuauuuUagu......................   2     1   Fm1
......................................................................aUgcaccaggcuauuucagu......................   1     1   Fm1
......................................................................aagcaccaggcuauuucGgu......................   2     1   Fm1
......................................................................aagcaccaggcuauuucaUu......................   6     1   Fm1
......................................................................aagcaccaggcuauuucagC......................   1     1   Fm1
......................................................................aagcaccaggcuauuuGagu......................   1     1   Fm1
......................................................................aagcaccaggcuauuuAagu......................   1     1   Fm1
......................................................................Uagcaccaggcuauuucagu......................   1     1   Fm1
......................................................................aagcaccaggcuauuucUgu......................   1     1   Fm1
......................................................................aagcaccaggcuauuucagCu.....................   1     1   Fm1
......................................................................aagcaccaggcuauuucaguG.....................   3     1   Fm1
......................................................................aagcaccaggcuauuuUaguu.....................   6     1   Fm1
......................................................................aaCcaccaggcuauuucaguu.....................   2     1   Fm1
......................................................................aagcaccaggcuauuucaguu.....................   2501  0   Fm1
......................................................................aagcaccaggcuauuuAaguu.....................   10    1   Fm1
......................................................................aagcaccGggcuauuucaguu.....................   1     1   Fm1
......................................................................aagcaccaggcAauuucaguu.....................   1     1   Fm1
......................................................................aagcacUaggcuauuucaguu.....................   1     1   Fm1
......................................................................aagcaccaggcuauuuGaguu.....................   5     1   Fm1
......................................................................aagcaccaggGuauuucaguu.....................   2     1   Fm1
......................................................................aagcaccaggcCauuucaguu.....................   1     1   Fm1
......................................................................aagcaccaggcuauuucaguA.....................   17    1   Fm1
......................................................................aagcaccaAgcuauuucaguu.....................   1     1   Fm1
```

Star Mature

agccagaacagagcugcugaaccuauccaguggacugggauuccuugugugcuguuugucauaucuuccaaagcaccaggcuauuucaguucucugguugggagaauucgac			
....aagcacGaggcuauuucaguu...................	1	1	Fm1
....aaUcaccaggcuauuucaguu...................	1	1	Fm1
....aagcaccaggUuauuucaguu...................	5	1	Fm1
....aagcaccaggcuauuCcaguu...................	2	1	Fm1
....aagcaccaggcuauuucGguu...................	9	1	Fm1
....aagcaccaggAuauuucaguu...................	7	1	Fm1
....aNgcaccaggcuauuucaguu...................	1	1	Fm1
....aagcaccaggcuauuucUguu...................	4	1	Fm1
....aagcaccaggcuauuucaguC...................	1	1	Fm1
....aagcGccaggcuauuucaguu...................	2	1	Fm1
....aagcaccaggcuauuucCguu...................	10	1	Fm1
....aagcUccaggcuauuucaguu...................	1	1	Fm1
....aGgcaccaggcuauuucaguu...................	1	1	Fm1
....aagcaccaggcuauuAcaguu...................	1	1	Fm1
....aagUaccaggcuauuucaguu...................	1	1	Fm1
....aagcaccaggcuaAuucaguu...................	1	1	Fm1
....aagcaccaggcuaGuucaguuc..................	6	1	Fm1
....aagcaccaggcuauuucaAuuc..................	3	1	Fm1
....Nagcaccaggcuauuucaguuc..................	26	1	Fm1
....aCgcaccaggcuauuucaguuc..................	2	1	Fm1
....aagcaccaggcuaCuucaguuc..................	10	1	Fm1
....aagcaccaggcuauuuAaguuc..................	224	1	Fm1
....aagcaccaggcuauuucagCuc..................	5	1	Fm1
....aagcaccaUgcuauuucaguuc..................	11	1	Fm1
....Uagcaccaggcuauuucaguuc..................	34	1	Fm1
....aagcacUaggcuauuucaguuc..................	22	1	Fm1
....aagcaccaggcuauuucaguuU..................	415	1	Fm1
....Cagcaccaggcuauuucaguuc..................	3	1	Fm1
....aUgcaccaggcuauuucaguuc..................	5	1	Fm1
....aagcaccaAgcuauuucaguuc..................	4	1	Fm1
....aagcaccGggcuauuucaguuc..................	14	1	Fm1
....aagcaccaggAuauuucaguuc..................	125	1	Fm1
....aagGaccaggcuauuucaguuc..................	1	1	Fm1
....aagcaccUggcuauuucaguuc..................	18	1	Fm1
....aagcaccaggcuauGucaguuc..................	3	1	Fm1
....aagcaccaggUuauuucaguuc..................	54	1	Fm1
....aagcaccaggcuauuucGguuc..................	172	1	Fm1
....aagcaccaggcuGuuucaguuc..................	2	1	Fm1
....aagcaccaggcuaAuucaguuc..................	7	1	Fm1
....aagcaccaggcuauuCcaguuc..................	12	1	Fm1
....aagcaccaggcuauuucaCuuc..................	4	1	Fm1
....aaAcaccaggcuauuucaguuc..................	7	1	Fm1
....aagcaccaggcuauuucCguuc..................	174	1	Fm1
....aagcaccagUcuauuucaguuc..................	10	1	Fm1
....aaNcaccaggcuauuucaguuc..................	1	1	Fm1
....aGgcaccaggcuauuucaguuc..................	15	1	Fm1
....aagcaccCggcuauuucaguuc..................	5	1	Fm1
....aagcaccaggcuauuucaguuc..................	59669	0	Fm1
....aagcacAaggcuauuucaguuc..................	4	1	Fm1
....aagcaccaggcuauuucaUuuc..................	9	1	Fm1
....aagcaccaCgcuauuucaguuc..................	3	1	Fm1
....aagUaccaggcuauuucaguuc..................	2	1	Fm1
....aaUcaccaggcuauuucaguuc..................	12	1	Fm1
....aagcaccaggcuauuucagAuc..................	4	1	Fm1
....aagcaccaggcuauuucUguuc..................	184	1	Fm1
....aagcaccaggcuauuucaguuG..................	6	1	Fm1
....aagcaccaggcuauuuGaguuc..................	75	1	Fm1
....aagcaccaggcuCuuucaguuc..................	3	1	Fm1
....aagcaAcaggcuauuucaguuc..................	9	1	Fm1
....aagcCccaggcuauuucaguuc..................	5	1	Fm1
....aagcaUcaggcuauuucaguuc..................	23	1	Fm1
....Gagcaccaggcuauuucaguuc..................	13	1	Fm1
....aagcaccaggcuauAucaguuc..................	5	1	Fm1
....aagcaccaggcAauuucaguuc..................	1	1	Fm1
....aagcaccaggcCauuucaguuc..................	6	1	Fm1
....aagcaccaggcuauuucaguAc..................	2	1	Fm1
....aagcaccaggcGauuucaguuc..................	2	1	Fm1
....aagcGccaggcuauuucaguuc..................	8	1	Fm1
....aagcaccaggcuauuuUaguuc..................	137	1	Fm1
....aagcaccaggcuauuAcaguuc..................	5	1	Fm1

Star Mature

agccagaacagagcugcugaaccuauccaguggacugggauuccuugugugcuguuugucauaucuuccaaagcaccaggcuauuucaguucucugguugggagaauucgac			
....aagcUccaggcuauuucaguuc........................	3	1	Fm1
....aagAaccaggcuauuucaguuc........................	5	1	Fm1
....aagcaccaggGuauuucaguuc........................	69	1	Fm1
....aagcaccaggcuauuucaguCc........................	11	1	Fm1
....aagcaGcaggcuauuucaguuc........................	3	1	Fm1
....aagcaccagCcuauuucaguuc........................	3	1	Fm1
....aagcacNaggcuauuucaguuc........................	5	1	Fm1
....aaCcaccaggcuauuucaguuc........................	11	1	Fm1
....aagcaccaggcuUuuucaguuc........................	4	1	Fm1
....aagcaccaggcuauuucaguuA........................	46	1	Fm1
....aagcaccaggcuauCucaguuc........................	8	1	Fm1
....aagcaccagAcuauuucaguuc........................	7	1	Fm1
....aagcaccaggcuauuucagGuc........................	3	1	Fm1
....aagcacGaggcuauuucaguuc........................	6	1	Fm1
....aagcaNcaggcuauuucaguuc........................	11	1	Fm1
....aagcaccaggcuauuucagAucu.......................	2	1	Fm1
....aagcaccaggcuauuucaguucC.......................	173	1	Fm1
....aagcaccaggcuauuuGaguucu.......................	2	1	Fm1
....aCgcaccaggcuauuucaguucu.......................	1	1	Fm1
....aaAcaccaggcuauuucaguucu.......................	1	1	Fm1
....aagcaccaggUuauuucaguucu.......................	9	1	Fm1
....aagcaccaggcuauuucGguucu.......................	11	1	Fm1
....aagcaccagUcuauuucaguucu.......................	1	1	Fm1
....aagcaccaggcuauuuUaguucu.......................	9	1	Fm1
....aagcUccaggcuauuucaguucu.......................	1	1	Fm1
....Nagcaccaggcuauuucaguucu.......................	3	1	Fm1
....aagcaccaggcuauuucaguuUu.......................	49	1	Fm1
....aagcaccaggcuauuucaUuucu.......................	1	1	Fm1
....aagcaccaggGuauuucaguucu.......................	4	1	Fm1
....aagcaccUggcuauuucaguucu.......................	1	1	Fm1
....aagcaccaggcuaGuucaguucu.......................	1	1	Fm1
....aagcaccaggcuauuucaguucG.......................	19	1	Fm1
....aagcaccaggcCauuucaguucu.......................	2	1	Fm1
....aagcaccaUgcuauuucaguucu.......................	1	1	Fm1
....aagcaccaggcuauuucaguuGu.......................	6	1	Fm1
....aagcaccaggcuauuucCguucu.......................	16	1	Fm1
....aagcaccaggcuauuucaguucA.......................	1764	1	Fm1
....aagcaUcaggcuauuucaguucu.......................	2	1	Fm1
....aaUcaccaggcuauuucaguucu.......................	2	1	Fm1
....aagcaccaggcuauuucaguucu.......................	4584	0	Fm1
....aagcaccaggcuauuuAaguucu.......................	17	1	Fm1
....aagAaccaggcuauuucaguucu.......................	1	1	Fm1
....Uagcaccaggcuauuucaguucu.......................	1	1	Fm1
....aagcaccaggcuauGucaguucu.......................	1	1	Fm1
....Gagcaccaggcuauuucaguucu.......................	2	1	Fm1
....aagcaccaggcuauuucagCucu.......................	4	1	Fm1
....aagcacAaggcuauuucaguucu.......................	1	1	Fm1
....aagcaccaggcuauuucUguucu.......................	13	1	Fm1
....aagcaccaggcuauuucaAuucu.......................	1	1	Fm1
....aagcaccaggAuauuucaguucu.......................	10	1	Fm1
....aagcaccaggcuauuucaguuAu.......................	12	1	Fm1
....aagGaccaggcuauuucaguucu.......................	3	1	Fm1
....aagcaccaggcuauuucaguCcu.......................	2	1	Fm1
....aagcaccaggcuauuucaguucuU......................	239	1	Fm1
....aagcaccaggcuauuucaguucAc......................	2	1	Fm1
....aagcaccaggcuauuucaguucuc......................	1	0	Fm1
....aagcaccaggcuauuucaguucuA......................	18	1	Fm1
....aagcaccaggcuauuucaguucuUu.....................	24	1	Fm1
....agcaccaggcuauuucaguuc........................	20	0	Fm1
....agcaccaggcuauuucagAuc........................	1	1	Fm1
....agcaccaggcuauuucaguucu.......................	1	0	Fm1
....caccaggcuauuucaguucucu.....................	3	0	Fm1
....aaagcaccaggcuauuuGaguu.........................	1	1	Fm2
....aaagcaccaggcuauuucaguu.........................	7	0	Fm2
....aaagcaccaggcuauuucaguuc........................	33	0	Fm2
....Caagcaccaggcuauuucaguuc........................	7	1	Fm2
....aagcaccaggcuauuuca............................	18	0	Fm2
....aagcaccaggGuauuucag...........................	1	1	Fm2
....aagcaccaggcuauuucag...........................	33	0	Fm2

Star Mature

agccagaacagagcugcugaaccuauccaguggacugggauuccuugugugcuguuugucauaucuuccaaagcaccaggcuauuucaguucucugguugggagaauucgac			
....aagcaccaggcuauuuGag.......................	1	1	Fm2
....aagcaccaggcuauuuAag.......................	1	1	Fm2
....aagcaccaggcuauuucGgu......................	1	1	Fm2
....aagcaccaggcuauuucUgu......................	1	1	Fm2
....Nagcaccaggcuauuucagu......................	1	1	Fm2
....aagcaccagUcuauuucagu......................	1	1	Fm2
....aagcaccaggcuauuuAagu......................	1	1	Fm2
....aagcaccaggcuauuucagu......................	155	0	Fm2
....aagcaccaggcuauuucCgu......................	1	1	Fm2
....aagcaccagCcuauuucaguu.....................	1	1	Fm2
....aagcaccaggcuauuucUguu.....................	3	1	Fm2
....aagcaccaggAuauuucaguu.....................	1	1	Fm2
....aagcaccaggcuauuuUaguu.....................	3	1	Fm2
....aagcaccaggcuauuuAaguu.....................	3	1	Fm2
....aagcaccaggcuauuucaguu.....................	680	0	Fm2
....aagcaccaggcuauuucGguu.....................	1	1	Fm2
....aGgcaccaggcuauuucaguu.....................	1	1	Fm2
....Uagcaccaggcuauuucaguu.....................	1	1	Fm2
....aagcaccaggGuauuucaguu.....................	1	1	Fm2
....aagcaccaggcuauuuGaguu.....................	1	1	Fm2
....aagcaccaggcuauuucCguu.....................	1	1	Fm2
....aagcaccaggGuauuucaguuc....................	28	1	Fm2
....aagcaAcaggcuauuucaguuc....................	1	1	Fm2
....aagcaccGggcuauuucaguuc....................	3	1	Fm2
....aagcaccaUgcuauuucaguuc....................	10	1	Fm2
....aagcaccaggcuauuAcaguuc....................	3	1	Fm2
....aagUaccaggcuauuucaguuc....................	1	1	Fm2
....aagcCccaggcuauuucaguuc....................	3	1	Fm2
....Cagcaccaggcuauuucaguuc....................	2	1	Fm2
....aagcaccaggcuauuucaguuA...................	25	1	Fm2
....aagcaccaAgcuauuucaguuc....................	1	1	Fm2
....aaUcaccaggcuauuucaguuc....................	8	1	Fm2
....aagcaccaggcCauuucaguuc....................	2	1	Fm2
....aagcaccaggcuCuuucaguuc....................	1	1	Fm2
....aagcacUaggcuauuucaguuc....................	4	1	Fm2
....aUgcaccaggcuauuucaguuc....................	1	1	Fm2
....aaAcaccaggcuauuucaguuc....................	8	1	Fm2
....aagcaccaggcuauuucaguuc....................	23857	0	Fm2
....aagcaccaggcuauuucCguuc....................	72	1	Fm2
....aagcaccUggcuauuucaguuc....................	5	1	Fm2
....aagcaccaggcuauuuUaguuc....................	58	1	Fm2
....aaNcaccaggcuauuucaguuc....................	1	1	Fm2
....aagcaccaggcuauuGcaguuc....................	1	1	Fm2
....aagcacAaggcuauuucaguuc....................	5	1	Fm2
....aagcaccaggcuauuuGaguuc....................	29	1	Fm2
....aagcaccCggcuauuucaguuc....................	1	1	Fm2
....aagcaGcaggcuauuucaguuc....................	1	1	Fm2
....aagGaccaggcuauuucaguuc....................	1	1	Fm2
....aagcaNcaggcuauuucaguuc....................	6	1	Fm2
....Nagcaccaggcuauuucaguuc....................	12	1	Fm2
....aGgcaccaggcuauuucaguuc....................	4	1	Fm2
....aagcaccagUcuauuucaguuc....................	4	1	Fm2
....aagcaccaggcuauuucGguuc....................	94	1	Fm2
....aagcaccaggcuaGuucaguuc....................	2	1	Fm2
....aagcaccaggcuUuuucaguuc....................	6	1	Fm2
....Uagcaccaggcuauuucaguuc....................	11	1	Fm2
....aagcaccaggUuauuucaguuc....................	21	1	Fm2
....aaCcaccaggcuauuucaguuc....................	3	1	Fm2
....aagcacNaggcuauuucaguuc....................	2	1	Fm2
....aagcaccaggcuauuucaUuuc....................	5	1	Fm2
....aagcUccaggcuauuucaguuc....................	5	1	Fm2
....aCgcaccaggcuauuucaguuc....................	5	1	Fm2
....aagcaccaggcuauAucaguuc....................	2	1	Fm2
....aagcaccaggAuauuucaguuc....................	44	1	Fm2
....aagcaccaggcuGuuucaguuc....................	3	1	Fm2
....aagcaccaggcuauuucaguuU...................	103	1	Fm2
....aagcaccaggcuauuucUguuc....................	74	1	Fm2
....aagcaccaggcuauuucaguCc....................	1	1	Fm2
....Gagcaccaggcuauuucaguuc....................	2	1	Fm2
....aagcacGaggcuauuucaguuc....................	2	1	Fm2

```
                                 Star                                 Mature
agccagaacagagcugcugaaccuauccaguggacugggauuccuugugugcuguuugucauaucuuccaaagcaccaggcuauuucaguucucugguugggagaauucgac
......................................................................aagAaccaggcuauuucaguuc....................    6       1    Fm2
......................................................................aagcaccaggcuaCuucaguuc....................    8       1    Fm2
......................................................................aagcaccaggcuauuucagAuc....................    1       1    Fm2
......................................................................aagcaccaggcAauuucaguuc....................    2       1    Fm2
......................................................................aagcaccagCcuauuucaguuc....................    2       1    Fm2
......................................................................aagcaccaggcuauuuAaguuc....................    110     1    Fm2
......................................................................aNgcaccaggcuauuucaguuc....................    1       1    Fm2
......................................................................aagcGccaggcuauuucaguuc....................    6       1    Fm2
......................................................................aagcaccagAcuauuucaguuc....................    3       1    Fm2
......................................................................aagcaUcaggcuauuucaguuc....................    6       1    Fm2
......................................................................aagcaccaggcuaAuucaguuc....................    9       1    Fm2
......................................................................aagcaccaggcuauuCcaguuc....................    7       1    Fm2
......................................................................aagcaccaggcuauuuUaguucu...................    2       1    Fm2
......................................................................aagcaccaggcuauuucaguucu...................    2071    0    Fm2
......................................................................aagcaccaggGuauuucaguucu...................    5       1    Fm2
......................................................................aaUcaccaggcuauuucaguucu...................    2       1    Fm2
......................................................................aagcaccaggcuauuuAaguucu...................    9       1    Fm2
......................................................................aagcaccaggUuauuucaguucu...................    1       1    Fm2
......................................................................aagcaccaggAuauuucaguucu...................    6       1    Fm2
......................................................................aagcaccaUgcuauuucaguucu...................    1       1    Fm2
......................................................................aagcaccaggcuauuucaguucA...................    1347    1    Fm2
......................................................................aagcaccaggcuauuucaguucG...................    9       1    Fm2
......................................................................aagcaAcaggcuauuucaguucu...................    1       1    Fm2
......................................................................aagcaccaggcuauuucaUuucu...................    1       1    Fm2
......................................................................Uagcaccaggcuauuucaguucu...................    2       1    Fm2
......................................................................aagcaUcaggcuauuucaguucu...................    1       1    Fm2
......................................................................aagcaccaggcuaAuucaguucu...................    1       1    Fm2
......................................................................aagcaccaggcuauuuGaguucu...................    2       1    Fm2
......................................................................aagcaccaggcuGuuucaguucu...................    1       1    Fm2
......................................................................aagcaccaggcuauuucGguucu...................    4       1    Fm2
......................................................................aagcaccaggcuauuucCguucu...................    6       1    Fm2
......................................................................aagcaccaggcuauuucaguucC...................    116     1    Fm2
......................................................................aagcaccaggcuauuucUguucu...................    5       1    Fm2
......................................................................aagcaccaggcuauuucaguucuU..................    118     1    Fm2
......................................................................aagcaccaggcuauuucaguucuUu.................    22      1    Fm2
.......................................................................agcaccaggcuauuucaguuc....................    8       0    Fm2

.gccagaacagagcugcugaaccuauccagugg...............................................................................    1       0    Mm3
........cagagcugcugaaccuauccagugg...............................................................................    1       0    Mm3
..............................uggacugggauuccuugugugcu...........................................................    7       0    Mm3
..............................uggacugggauuccuugugugcuU..........................................................    1       1    Mm3
.................................acugggauuccuugugugcug..........................................................    3       0    Mm3
.................................acugggauuccuugugugcugu.........................................................    2       0    Mm3
.................................acugggauuccuugugugcuguu........................................................    8       0    Mm3
.................................acugggauuccuugugugcuguuu.......................................................    8       0    Mm3
.................................acugggauuccuugugugcuguuug......................................................    3       0    Mm3
.................................acugggauuccuugugugcuguuuguc....................................................    2       0    Mm3
.............................................ugugugcuguuugucauaucuucca..........................................    1       0    Mm3
........................................................ugucauaucuuccaaagcaccaggcu..............................    1       0    Mm3
..........................................................ucauaucuuccaaagcaccaggc...............................    2       0    Mm3
..........................................................ucauaucuuccaaagcaccaggcu..............................    4       0    Mm3
..........................................................ucauaucuuccaaagcaccaggcuauuucaguuc....................    1       0    Mm3
..........................................................ucauaucuuccaaagcaccaggcuauuucaguucu...................    1       0    Mm3
.....................................................................aaagcaccaggcuauuucaguu.....................    2       0    Mm3
.....................................................................aaagcaccaggcuauuucaguuc....................    4       0    Mm3
.....................................................................Caagcaccaggcuauuucaguuc....................    2       1    Mm3
......................................................................aagcaccaggcuauuuca........................    22      0    Mm3
......................................................................aagcaccaggcuauuucag.......................    16      0    Mm3
......................................................................aagcaccaggcuauuucaA.......................    1       1    Mm3
......................................................................aagcaccaggcuauuucagu......................    82      0    Mm3
......................................................................aagcaccaggUuauuucagu......................    1       1    Mm3
......................................................................Uagcaccaggcuauuucaguu.....................    1       1    Mm3
......................................................................aagcaccaggcuauuucaguA.....................    1       1    Mm3
......................................................................aagcaccaggcuauuucaguu.....................    354     0    Mm3
......................................................................aagcaccaggcuauuucUguu.....................    4       1    Mm3
......................................................................aagcaccaggcuauuuAaguu.....................    1       1    Mm3
......................................................................aagcGccaggcuauuucaguu.....................    1       1    Mm3
......................................................................aagcaccaggcuauuuUaguu.....................    1       1    Mm3
......................................................................aagcacUaggcuauuucaguu.....................    1       1    Mm3
......................................................................aagcaccUggcuauuucaguuc....................    2       1    Mm3
```

Star Mature

```
agccagaacagagcugcugaaccuauccaguggacugggauuccuugugugcuguuugucauaucuuccaaagcaccaggcuauuucaguucucugguugggagaauucgac
......................................................................Gagcaccaggcuauuucaguuc....................  1     1   Mm3
......................................................................aagcaccaUgcuauuucaguuc....................  2     1   Mm3
......................................................................aagcaccaggGuauuucaguuc....................  8     1   Mm3
......................................................................aagcaccagAcuauuucaguuc....................  2     1   Mm3
......................................................................aagcaccaggcuUuuucaguuc....................  2     1   Mm3
......................................................................aagcaccaggcuauuucaguuc....................  5522  0   Mm3
......................................................................aagcaccaggcuauuucaguuA....................  10    1   Mm3
......................................................................aagcaccaggcuauuucGguuc....................  20    1   Mm3
......................................................................aCgcaccaggcuauuucaguuc....................  2     1   Mm3
......................................................................aagcaccaggcuauuucagGuc....................  1     1   Mm3
......................................................................aagcaccCggcuauuucaguuc....................  1     1   Mm3
......................................................................aagcaccaggcuauuucUguuc....................  14    1   Mm3
......................................................................aaUcaccaggcuauuucaguuc....................  2     1   Mm3
......................................................................Nagcaccaggcuauuucaguuc....................  6     1   Mm3
......................................................................aagcaccaggAuauuucaguuc....................  6     1   Mm3
......................................................................aagcaUcaggcuauuucaguuc....................  2     1   Mm3
......................................................................aagcaccaggcuauuCcaguuc....................  1     1   Mm3
......................................................................aagcaccaggcuauuuUaguuc....................  20    1   Mm3
......................................................................aagcaccaggcuauuuAaguuc....................  25    1   Mm3
......................................................................aagcaccaggcuGuuucaguuc....................  1     1   Mm3
......................................................................aagcaccaggcuauuucaUuuc....................  2     1   Mm3
......................................................................aaAcaccaggcuauuucaguuc....................  3     1   Mm3
......................................................................aagcaccaggcuauuucCguuc....................  17    1   Mm3
......................................................................aagcaccaggUuauuucaguuc....................  4     1   Mm3
......................................................................aagcaccaggcuaAuucaguuc....................  1     1   Mm3
......................................................................aagcaccaggcuauuucaguAc....................  2     1   Mm3
......................................................................aagcaNcaggcuauuucaguuc....................  1     1   Mm3
......................................................................aGgcaccaggcuauuucaguuc....................  1     1   Mm3
......................................................................aagcaccGggcuauuucaguuc....................  1     1   Mm3
......................................................................aagcaccaggcuauuuGaguuc....................  4     1   Mm3
......................................................................aagcaccaggcuauuucaguuU....................  36    1   Mm3
......................................................................aagcaccaggcuauuucaguCc....................  2     1   Mm3
......................................................................aagcaAcaggcuauuucaguuc....................  1     1   Mm3
......................................................................aagcacUaggcuauuucaguuc....................  4     1   Mm3
......................................................................Uagcaccaggcuauuucaguuc....................  3     1   Mm3
......................................................................aagcaccaggcuauuucaguucu...................  444   0   Mm3
......................................................................aagcaccaggcuauuucaguucC...................  5     1   Mm3
......................................................................aagcaccaggcuauuucaguuAu...................  1     1   Mm3
......................................................................aagcaccaggGuauuucaguucu...................  1     1   Mm3
......................................................................aagcaccaggcuauuuAaguucu...................  1     1   Mm3
......................................................................aaCcaccaggcuauuucaguucu...................  1     1   Mm3
......................................................................aagcaNcaggcuauuucaguucu...................  1     1   Mm3
......................................................................aagcaccaggcuauuucCguucu...................  1     1   Mm3
......................................................................aagcaccaggcuauuuGaguucu...................  1     1   Mm3
......................................................................aagcaccaggcuauuucUguucu...................  1     1   Mm3
......................................................................aagcaccaggcuauuucaguucA...................  237   1   Mm3
......................................................................aagcaccaggcuauuuUaguucu...................  1     1   Mm3
......................................................................aagcacUaggcuauuucaguucu...................  1     1   Mm3
......................................................................aagUaccaggcuauuucaguucu...................  1     1   Mm3
......................................................................aagcaccaggcuauuucGguucu...................  2     1   Mm3
......................................................................aagcaccaggcuauuucaguuUu...................  2     1   Mm3
......................................................................aagcaccaggcuauuucaguucG...................  1     1   Mm3
......................................................................aagcaccaggcuauuucaguucuU..................  23    1   Mm3
......................................................................aagcaccaggcuauuucaguucuA..................  2     1   Mm3
......................................................................aagcaccaggcuauuucaguucuUu.................  5     1   Mm3
.......................................................................agcaccaggcuauuucaguu.....................  1     0   Mm3
.......................................................................agcaccaggcuauuucaguuc....................  5     0   Mm3
.......................................................................agcaccaggcuauuucaguucu...................  1     0   Mm3
........................................................................gcaccaggcuauuucaguuc....................  1     0   Mm3

...........agcugcugaaccuauccagugg...............................................................................  1     0   Fm3
..............................uggacugggUuuccuugugugcu...........................................................  1     1   Fm3
..............................uggacugggauuccuugugugcu...........................................................  30    0   Fm3
.................................acugggauuccuugugAgcug..........................................................  1     1   Fm3
.................................acugggauuccuugugCgcug..........................................................  1     1   Fm3
.................................acugggauuccuuguguUcug..........................................................  2     1   Fm3
.................................acugggauuccuugugugcug..........................................................  98    0   Fm3
.................................acugggauuccuugugugcugu.........................................................  9     0   Fm3
.................................acugggauuccuugugugcuguu........................................................  9     0   Fm3
.................................acugggauuccuugugugcuguuu.......................................................  1     0   Fm3
```

Star Mature

agccagaacagagcugcugaaccuauccaguggacugggauuccuugugugcuguuugucauaucuuccaaagcaccaggcuauuucaguucucugguugggagaauucgac			
.................................acugggauuccuugugugcuguuug..	4	0	Fm3
....aaagcaccaggcuauuucag.......................	2	0	Fm3
....aaagcaccaggcuauuucaguuc....................	1	0	Fm3
....aagcaccaggcuauuuca........................	30	0	Fm3
....aagcaccaggcuauuucU........................	2	1	Fm3
....aagcaccaggcuauuucUg.......................	1	1	Fm3
....aagcaccaggcuauuucag.......................	678	0	Fm3
....aagcaccaggcuauuucGg.......................	3	1	Fm3
....aagcaccaggcuauuucaA.......................	3	1	Fm3
....aagcaccGggcuauuucag.......................	1	1	Fm3
....aagcaccaggAuauuucag.......................	1	1	Fm3
....aagcaccaggcuauuuAag.......................	4	1	Fm3
....aagcacAaggcuauuucag.......................	1	1	Fm3
....aagcaccaggcuauuuUag.......................	3	1	Fm3
....aagcaccaggcuauuucagu......................	141	0	Fm3
....aagcaccaggcuauuucUgu......................	1	1	Fm3
....aagcaccaggcuauuucagA......................	1	1	Fm3
....aagcaccaggcuaCuucagu......................	1	1	Fm3
....aagcaccaggcuauuucGgu......................	1	1	Fm3
....aagcaccaggcuauuuAaguu.....................	1	1	Fm3
....aagcaccaggcuauuucaguu.....................	401	0	Fm3
....aagcaccaggcuauuucGguu.....................	1	1	Fm3
....aaUcaccaggcuauuucaguu.....................	1	1	Fm3
....aagcaccaggAuauuucaguu.....................	1	1	Fm3
....aagcaccaggcuUuuucaguu.....................	1	1	Fm3
....aagcaccaggcuauuucaguA.....................	2	1	Fm3
....aagcaccaggcuauuuGaguu.....................	2	1	Fm3
....aagcaccaggcuauuuUaguu.....................	4	1	Fm3
....aagcaccaggcuauuucCguu.....................	1	1	Fm3
....aagcaccaggcuauuucUguu.....................	2	1	Fm3
....aagcaccaUgcuauuucaguuc....................	1	1	Fm3
....aagcaccaggcuauuucCguuc....................	4	1	Fm3
....aagcaccaggcuaAuucaguuc....................	1	1	Fm3
....aagcacUaggcuauuucaguuc....................	1	1	Fm3
....aagcaccaggcuUuuucaguuc....................	1	1	Fm3
....Gagcaccaggcuauuucaguuc....................	1	1	Fm3
....aagcaccaggcuauCucaguuc....................	3	1	Fm3
....aGgcaccaggcuauuucaguuc....................	1	1	Fm3
....aagcaccaggcuauuucUguuc....................	6	1	Fm3
....aagcaccaggUuauuucaguuc....................	7	1	Fm3
....aagcaccaggAuauuucaguuc....................	5	1	Fm3
....aagcaccaggcuauuuAaguuc....................	8	1	Fm3
....aagcaccaggcuauuCcaguuc....................	1	1	Fm3
....aagcUccaggcuauuucaguuc....................	1	1	Fm3
....aagcaccaggcuauuucaguuG....................	1	1	Fm3
....Nagcaccaggcuauuucaguuc....................	1	1	Fm3
....aagcaccaggcuauuuUaguuc....................	6	1	Fm3
....Uagcaccaggcuauuucaguuc....................	2	1	Fm3
....aagGaccaggcuauuucaguuc....................	1	1	Fm3
....aagcaccaggcuauuucaguAc....................	2	1	Fm3
....aagcaccaggcuauuucGguuc....................	6	1	Fm3
....aagcaccaggcuauuucaguuU....................	20	1	Fm3
....aagcaccaggcuauuuGaguuc....................	3	1	Fm3
....aagcaccaggGuauuucaguuc....................	5	1	Fm3
....aagcaccaggcuauuucaguuA....................	7	1	Fm3
....aagcaUcaggcuauuucaguuc....................	2	1	Fm3
....aaUcaccaggcuauuucaguuc....................	2	1	Fm3
....aagcaccaggcuauuucaguuc....................	2549	0	Fm3
....aagcaccaggcuauCucaguucu...................	1	1	Fm3
....aagcaccaggcuauuucaguuUu...................	1	1	Fm3
....aagcaccaggcuauuucaguucu...................	167	0	Fm3
....aaAcaccaggcuauuucaguucu...................	1	1	Fm3
....aagcaccaggcuauuucaguucC...................	7	1	Fm3
....aagcaccaggcuauuucaguucA...................	119	1	Fm3
....aagcaccaggcuauuucaguucuU..................	1	1	Fm3
....aagcaccaggcuauuucaguucuA..................	1	1	Fm3
....agcaccaggcuauuucaguu.....................	1	0	Fm3
....agcaccaggcuauuucaguuc....................	8	0	Fm3
....agcaccaggcuauuucaguucu...................	1	0	Fm3
